# Supplementary material for: NEDD4L binds the proteasome and promotes autophagy and bortezomib sensitivity in multiple myeloma
Source: Cell Death Dis. 2022 Mar 2;13(3):197. doi: 10.1038/s41419-022-04629-8 (PMC8891287; doi:10.1038/s41419-022-04629-8)
Supplement: Supplementary file 1 — the copy of the revised article highlighted changes [file 41419_2022_4629_MOESM1_ESM.docx]

**Title**: NEDD4L binds the proteasome and promotes autophagy and bortezomib sensitivity in multiple myeloma

**Running Title:** NEDD4L in multiple myeloma

**Author list**: Xi Huang^1*^, Wen Cao^1*^, Shunnan Yao^1^, Jing Chen^1^, Yang Liu^1^, Jianwei Qu^1^, Yi Li^1^, Xiaoyan Han^1^, Jingsong He^1^, He Huang^1,2^, Enfan Zhang^1#^, and Zhen Cai^1,2#^

* XH and WC contributed equally to this work

^#^ EFZ and ZC are co-corresponding authors

**Authors' Affiliation**: ^1^Bone Marrow Transplantation Center, Department of Hematology, The First Affiliated Hospital, School of Medicine, Zhejiang University, Hangzhou, China; ^2^Institute of Hematology, Zhejiang University, China.

**Corresponding Author**: Zhen Cai and Enfan Zhang, Bone Marrow Transplantation Center, Department of Hematology, The First Affiliated Hospital, School of Medicine, Zhejiang University, No. 79, Qingchun Road, Hangzhou, Zhejiang Province, China. Phone: 0086-571-87236706; Fax: 0086-571-87236706; E-mail: caiz@zju.edu.cn (ZC) and efzhang@zju.edu.cn (EFZ)

**Key words:** NEDD4L, bortezomib-resistance, autophagy, proteasome, myeloma

Word counts for text (excluding abstract, figures and tables, references, and methods.): 3307

Word counts for abstract: 150

Figure count: 6

Reference count: 40

**NEDD4L binds the proteasome and promotes autophagy and bortezomib sensitivity in multiple myeloma**

**Abstract**

Multiple myeloma (MM) remains an incurable plasma cell cancer characterized by abnormal secretion of monoclonal immunoglobulins. The molecular mechanism that regulates the drug sensitivity of MM cells is being intensively studied. Here, we report an unexpected finding that the protein encoded by neural precursor cell-expressed developmentally downregulated gene 4L (NEDD4L), which is a HECT E3 ligase, binds the 19S proteasome, limiting its proteolytic function and enhancing autophagy. Suppression of NEDD4L expression reduced bortezomib (Bor) sensitivity in vitro and in vivo, mainly through autophagy inhibition mediated by low NEDD4L expression, which was rescued by an autophagy activator. Clinically, elevated expression of NEDD4L is associated with a considerably increased probability of responding to Bor, a prolonged response duration, and improved overall prognosis, supporting both the use of NEDD4L as a biomarker to identify patients most likely to benefit from Bor and the regulation of NEDD4L as a new approach in myeloma therapy.

**Key points**

NEDD4L is a tumor suppressor in MM and sensitizes MM cells to Bor by promoting autophagy.

NEDD4L interacts with the proteasome and limits proteolytic function.

**Introduction**

Multiple myeloma (MM) is an incurable malignant proliferative disease of plasma cells. The main clinical manifestations of MM are hypercalcemia, renal insufficiency, anemia, and bone lesions (CRAB)^1^. The current treatment methods for MM include mainly proteasome inhibitors (PIs) and/or immunomodulatory drugs (IMiDs), CAR-T-cell therapy, and autologous hematopoietic stem cell transplantation (ASCT)^2^. Novel agents and combination regimens have shifted the perception of MM from a fatal, incurable disease to a chronic, manageable disease. Currently, the first-in-class PI bortezomib (Bor) is the backbone of MM therapy, but patients eventually develop secondary resistance despite improvements in the response depth and duration and prolonged survival^3^. Thus, from a clinical perspective, the identification of effective targets is urgently needed to solve the problem of Bor resistance.

MM cells and their normal counterparts secrete large amounts of immunoglobulins (Igs), and protein degradation in cells is carried out mainly by the ubiquitin-proteasome system (UPS) and the lysosomal pathway^4,5^. PIs have been reported to induce apoptosis in myeloma cells by disrupting the unfolded protein response (UPR)^6,7^. The balance between the intracellular protein load and proteasome capacity partially determines Bor sensitivity^8^. The 26s proteasome includes the core 20S proteasome (ex, PSMA5, PSMA2 and PSMB5) and the regulatory 19S (ex, PSMD2, PSMD4 and PSMC3), which can degrade ubiquitin-labeled substrates. (**Answers to Reviewer #2 Q4**) Mutation of the proteasome β5 subunit (PSMB5) has been described as one of the underlying mechanisms of Bor resistance^9,10^. Vogl *et al.* reported a phase I trial evaluating the safety and preliminary efficacy of separately targeting proteasomal and autophagic protein degradation using Bor and hydroxychloroquine in patients with relapsed or refractory MM^11^.

Neural precursor cell-expressed developmentally downregulated gene 4L (NEDD4L, also called NEDD4-2), which is identified as a HECT-type E3 ubiquitin ligase, is involved in the regulation of multiple substrates and mediation of drug sensitivity in diverse cancers^12^. In addition, recent studies found that an increase in NEDD4L expression coincides with UPR and autophagy activation caused by pharmaceuticals that induce ER stress^13^. Furthermore, NEDD4L ubiquitinates and degrades ULK1 kinase, which is an upstream player in autophagy responsible for the deactivation of autophagy^14^.

The NEDD4 family includes nine members, among which NEDD4-1 (NEDD4) and NEDD4-2 are the most closely related^15^. We previously provided evidence that NEDD4-1 overexpression sensitizes MM cells to Bor by Akt ubiquitination^16^. This study aimed to explore the relationship between NEDD4L and MM, the key signaling pathways regulated by NEDD4L and the possible implications of such regulation in MM.

**Materials and methods**

**Cell lines and chemicals**

The human myeloma cell lines (HMCLs) RPMI8226 and MM.1S were obtained from the Cell Bank of the Chinese Academy of Sciences. NCI-H929, ARP-1, CAG, OPM2, and RPMI8226.BR and ARK cells were gifted by Dr. Qing Yi (Center for Hematologic Malignancy Research Institute, Houston Methodist, USA). All human cell lines have been authenticated using STR profiling and all experiments were performed with mycoplasma-free cells. BM samples from MM patients and peripheral blood mononuclear cells (PBMCs) from healthy donors were obtained after informed consent was provided following approval by the Ethics Committee of the First Affiliated Hospital, College of Medicine, Zhejiang University. HMCLs were cultured in RPMI-1640 medium (Corning Cellgro, USA) supplemented with 10% fetal bovine serum (Thermo Fisher Scientific, Gibco, USA) at 37°C in a humidified atmosphere containing 5% CO_2_. Bortezomib, carfilzomib (CFZ), ixazomib (IXA), doxorubicin (ADM), melphalan (MEL), lenalidomide (LEN), rapamycin (Rapa), hydroxychloroquine sulfate (HCQ), Z-VAD-FMK, and NQDI-1 were purchased from Selleck Chemicals, LLC (Houston, TX, USA). The Bax inhibitor peptide V5 (Baxi) was obtained from MedChemExpress (New Jersey, USA).

**RNA interference**

A lentivirus containing green fluorescent protein (GFP) with a short hairpin RNA (shRNA) against human NEDD4L and a control lentivirus (NC-NEDD4L) were obtained from GeneChem (Shanghai, China) and transfected into MM cells according to the manufacturer’s instructions. Transfected cells were screened with puromycin (2 μg/ml). The NEDD4L shRNA GFP^+^ cell lines adopted in this manuscript were mixed populations. The siRNA oligo used to knock down NEDD4L was synthesized by Invitrogen (Life Technologies, USA). The sequence of the siRNA was as follows: NEDD4L, 5′-GAAGAGUUGCUGGUCUGGCCGUAUU-3′.

**Immunofluorescence and** **immunohistochemical analyses**

Paraformaldehyde-fixed, Triton X-100-permeabilized cells isolated from bone marrow (BM) biopsy tissues from MM patients were subjected to immunofluorescence staining to analyze the expression and localization of NEDD4L in CD138^+^ MM cells. Immunofluorescence analyses were also used to determine the level of LC3B (Sigma, St. Louis, MO, USA) in NEDD4L-knockdown (KD) cells. Additionally, paraformaldehyde-fixed, paraffin-embedded sections (5 μm) of tumor tissues from tumor-bearing nonobese diabetic-severe combined immunodeficiency (NOD-SCID) mice and BM biopsy tissues from MM patients were subjected to immunohistochemical staining to analyze NEDD4L, Ki67, and TUNEL. The data were analyzed using QuantCenter, Pannoramic Viewer (3D HISTECH, Hungary) and Image-Pro Plus 6.0 (Media Cybernetics, Inc., Rockville, MD, USA).

**Quantitative RT–PCR, western blotting and** **coimmunoprecipitation assays**

RNA and supernatants containing cellular protein were extracted from cells, and quantitative RT–PCR and western blotting were performed as previously described^16^. For co-immunoprecipitation (IP) assays, to immunoprecipitate endogenous proteins, whole-cell extracts were pretreated with protein A and G beads (Life Technologies, USA) and then incubated at 4°C with IgG, NEDD4L or PSMD2 overnight. After washing the beads three times with lysis buffer, the obtained immunoprecipitation complexes were subjected to SDS–PAGE analysis. Protein levels were analyzed with Image Lab software (Bio-Rad, USA), and the results are shown in SupFigs 3-5. The following primers were used for qRT-PCR: NEDD4L (forward 5’-GACATGGAGCATGGATGGGAA-3’; reverse 5’-GTTCGGCCTAAATTGTCCACT-3’) and GAPDH (forward, 5’-TTGGTATCGTGGAAGGACTCA-3’; reverse, 5’-TGTCATCATATTTGGCAGGTTT-3’). The primary antibodies and their sources were as follows: anti-NEDD4L, anti-PSMG1 (Abcam, Cambridge, UK), anti-GAPDH, anti-Lamin B1, anti-cleaved Caspase-3, anti-P21, anti-Ubiquitin, anti-LC3A/B, anti-mTOR, anti-phospho-mTOR (Ser2448) (P-mTOR), anti-PI3K, anti-AKT, anti-phospho-AKT (Ser473) (P-AKT), anti-PSMB5, anti-PSMA2, anti-PSMC3, anti-IκBα, anti-phospho-IκBα (Ser32) (P-IκBα) (Cell Signaling Technology, Danvers, USA), anti-PSMD2, anti-PA28α, anti-PSMA5, and anti-PSMD4 (Santa Cruz, Texas, USA). The Dynabeads® Co-Immunoprecipitation Kit was purchased from Thermo Fisher Scientific Inc.

**Cell proliferation and proteasome activity assays**

A cell counting kit-8 (CCK-8) assay (Dojindo, Japan) was used to evaluate MM cell proliferation and viability. MM cells (5× 10^4^ cells/ml) were plated in 24-well plates and then treated under the indicated conditions at 37°C in a humidified atmosphere containing 5% CO_2_. After 1–5 days, the cells were transferred to 96-well plates (100 μl/well) and treated with 10 μl of CCK-8 reagent. The 96-well plates were incubated at 37°C for 1–2 h. The absorbance was measured at 450 nm using a microplate reader (Bio-Rad, Model 680, USA). Cell viability was calculated as follows: Cell viability (%) = OD value of the test sample/OD value of the control sample × 100.

An in vitro proteasome activity assay was performed using SUC-LLVY-AMC (Enzo Life Sciences). (**Answers to Reviewer #2 Q1**) MM cells were harvested and washed twice with cold PBS, and total protein was extracted with lysis buffer (50 mM HEPES (pH 7.5), 5 mM EDTA, 150 mM NaCl, and 1% Triton X-100). After three freeze-thaw cycles, the samples were centrifuged at 1,200 rpm for 20 min at 4°C. The supernatants were collected and quantified with a BCA Protein Quantitation Assay Kit (KeyGen Biotech, China). Each sample consisted of 100 µl of reaction solution (25 mM HEPES (pH 7.5), 0.5 mM EDTA, 0.05% NP-40, and 0.001% SDS) containing 10 µg of protein and the fluorescent peptide substrate Suc-LLVY- AMC at a final concentration of 50 µM. The samples were incubated at 37°C. The experiment also included a positive control (1 μM MG132). After the substrate was hydrolyzed, the fluorescence intensity was measured at an excitation wavelength of 380 nM and emission wavelength of 460 nM using a Varioskan Flash instrument (Thermo Fisher Scientific, USA).

**Flow cytometry: Apoptosis, cell cycle and autophagy assays**

Apoptosis and cell cycle assays were conducted as previously described^16^. ARP-1 cells (1 × 10^5^ cells/ml) were treated with HCQ (10 μM) in 24-well plates for 24 h. The cells were harvested, and autophagy was detected by flow cytometry with an Enzo Cyto-ID Autophagy Detection Kit (Enzo Life Sciences, NY, USA) according to the manufacturer’s instructions.

**Animal studies**

All animal studies followed the procedures and protocols of the Animal Ethical Committee of the First Affiliated Hospital, College of Medicine, Zhejiang University. Four-week-old NOD-SCID male mice were used. The mice were injected subcutaneously in the left flank with 4 × 10^6^ ARP-1 cells resuspended in 50 μl of RPMI-1640 medium. When the established tumors reached approximately 100-130 mm^3^, the mice were randomly divided into 6 groups (n=3×6) and then received intraperitoneal injections of PBS or Bor (0.5 mg/kg, every 3-4 days). Tumor diameters were measured with calipers when Bor or PBS was injected, and tumor volumes were calculated as follows: 4π/3 × (a/2)^2^ × b/2, where a is the tumor width and b is the tumor length. The mice were then photographed and sacrificed. The tumor tissues of tumor-bearing NOD-SCID mice were harvested and used for immunohistochemical analyses as described above.

**Statistical analyses**

Data from two groups were analyzed by two-tailed Student’s t-tests in GraphPad Prism 6.0 (GraphPad Software, San Diego, CA, USA). The results are expressed as the means ± standard deviations (S.D.s) from three independent assays (*P < 0.05, **P < 0.01, and ***P < 0.001). At least three samples were chose to performed the experiments. For the original data, please contact caiz@zju.edu.cn.

**Results**

**Clinical significance of NEDD4L expression in MM**

To identify the significantly differentially expressed genes involved in the modulation of Bor resistance in MM, an Affymetrix HTA 2.0 array was employed to compare changes in gene expression levels between the HMCL RPMI8226 and Bor-resistant RPMI8226.BR cell lines^17^. We selected E3s among the differentially expressed genes because E3s are largely responsible for determining the spatiotemporal regulation of substrates in the UPS^18^. The expression levels of the top 14 differentially expressed E3s are shown in Figure 1A. We also verified some E3s by RT–PCR and found that NEDD4L was greatly downregulated in the Bor-resistant cell line compared with the control cells and played key roles in other tumors (SupFig 1A).

We analyzed independent MM-related microarray datasets from Oncomine (available online). NEDD4L gene expression based on 3 databases exhibited five results with a significant p-value (p < 0.001) and gene ranks in the top 10% among all differentially expressed genes. Data from Mulligan *et al*. showed that Bor nonresponders had significantly lower NEDD4L expression than Bor responders (Figure 1B). Patients with progression had lower expression of NEDD4L than nonprogressing patients (Figure 1C). In addition, data from Agnelli *et al*. indicated that the expression of NEDD4L was gradually reduced from normal plasma cells (NPCs) to monoclonal gammopathy of undetermined significance (MGUS) to MM to plasma cell leukemia (PCL) (Figure 1D). Moreover, patients with low expression of NEDD4L had more symptoms of bone lesions (SupFig 1B). Using data from Carrasco *et al*., we showed that MM patients with an “alive” overall survival status had significantly higher NEDD4L expression than those with a “dead” overall survival status (Figure 1E). Compared with patients without recurrence, patients with recurrence had decreased expression of NEDD4L (Figure 1F). Similarly, our analysis of NEDD4L showed that BM from relapsed/refractory MM patients (RRMM) had decreased NEDD4L expression compared with BM from healthy donors (HD) (Figure 1G), and the mRNA levels of NEDD4L were higher in new diagnosis MM (NDMM) than RRMM (Figure 1H). (**Answers to Reviewer #2 Q2**) Overall, the data from the above analyses strongly suggested that low NEDD4L expression by malignant plasma cells may be a risk factor in MM.

**NEDD4L expression in MM**

Next, we examined NEDD4L expression in HMCLs. Western bloting and RT–qPCR analyses indicated that different HMCLs expressed different levels of NEDD4L. PBMCs from healthy donors had relatively higher expression levels of NEDD4-1 mRNA and protein than RPMI8226 and ARP-1 (Figure 2A). To observe the localization of NEDD4L, we performed immunofluorescence assays. Figure 2B shows that NEDD4L was widely distributed in CD138^+^ primary MM cells and that its expression was higher in the cytoplasm than in the nucleus (data from the negative control and IgG control are shown in SupFig 1C).

To explore the relationship between the Bor concentration and NEDD4L expression, we treated ARP-1 and RPMI8226 cells with serially increasing concentrations of Bor. Western blotting and RT–qPCR results showed that Bor decreased NEDD4L expression in whole-cell lysates of HMCLs in a dose-dependent manner (Figure 2C). This phenomenon was equivalent in both the cytoplasm and nucleus, indicating that Bor did not change the localization of NEDD4L (Figure 2D). As reports indicate that NEDD4-1 is cleaved during apoptosis and that this cleavage is inhibited by an inhibitor of caspase-3-like proteases^19^, we assessed NEDD4L expression in the presence of Bor and the pancaspase inhibitor Z-VAD-FMK. However, NEDD4L expression was not rescued by Z-VAD-FMK (SupFig 1D). Furthermore, we also applied other apoptosis inhibitors, such as NQDI-1 and Baxi. Surprisingly, the level of NEDD4L did not decrease in HMCLs incubated with Bor in the presence of the apoptosis signal-regulated kinase 1 (ASK1) inhibitor NQDI (Figure 2E). Taken together, our results indicated that MM cells expressed low levels of NEDD4L and that NEDD4L was mostly expressed in the cytoplasm. NEDD4L expression was decreased under Bor treatment and could be rescued by NQDI-1.

**NEDD4L inhibition potentiates MM cell proliferation, Bor resistance and G2/M phase arrest in vitro**

To determine the effect of NEDD4L inhibition on MM cells, we knocked down NEDD4L by infecting MM cells with a lentivirus expressing three independent NEDD4L shRNAs plus GFP and selected GFP^+^-stable cell lines. We chose 6 or 7 sequences for subsequent studies because of the valid, consistent reduction in NEDD4L expression without an influence on the level of NEDD4-1 expression (Figure 3A). First, the cell growth curves showed that NEDD4L suppression promoted MM cell proliferation compared with that of control cells, and the differences increased in both the Bor-treated and 1% FBS-treated (serum reduction-induced) groups (Figure 3B). Second, the CCK-8 assay results indicated that knocking down NEDD4L dose-dependently attenuated the anti-MM efficacy of Bor in ARP-1 and RPMI8226 cells (Figure 3C). Third, we performed flow cytometry to assess apoptosis in HMCLs, with NEDD4L inhibition and control HMCLs treated with different concentrations of Bor for 24 h. The data in Figure 3D and SupFig 2A show that Bor-induced apoptosis in MM cells was significantly decreased when NEDD4L expression was suppressed and that the differences in both cell viability and apoptosis increased in the presence of Bor. In contrast, NEDD4L-overexpressing MM cells were more sensitive to Bor, exhibiting slower proliferation and increased apoptosis under Bor treatment (Figure 3E and SupFig 2B). Interestingly, we found that NEDD4L-KD MM cell lines were also more resistant to other PIs, including the clinically used carfilzomib and ixazomib (SupFig 2C and 2D). However, shRNA-transfected MM cells showed no obvious increases in viability compared with that of control cells when exposed to other classical drugs used to treat myeloma, such as lenalidomide, doxorubicin or melphalan (SupFig 2E and 2F). Fourth, flow cytometry was performed to evaluate the cell cycle in MM cells. As shown in Figure 3F, MM cells with NEDD4L KD were arrested in G2/M phase when treated with certain concentrations of Bor (27.39% vs. 48.98% vs. 46.32% for ARP-1 cells, p<0.05; 38.84% vs. 55.27% vs. 55.88% for RPMI8226 cells, p<0.05). Finally, western blotting was performed to verify the above results. Consistently, the levels of the cell cycle-related protein P21 were decreased in MM cells transfected with NEDD4L shRNAs (Figure 3G). These data indicated that NEDD4L might be a promising target in MM therapy.

**Low NEDD4L enhances Bor resistance through autophagy inhibition**

As mentioned above, autophagy plays an important role in the cytotoxicity of Bor to MM cells, and we examined the underlying mechanism of autophagy in NEDD4L-mediated resistance. First, we explored whether NEDD4L regulates autophagy. It has been reported that NEDD4L exerts a tumor-suppressive role by promoting PI3K ubiquitination and downregulation^20^. We also consistently found that NEDD4L silencing activated the PI3K/AKT/mTOR pathway (Figure 4A). Next, we performed western blot analysis to determine the effects of NEDD4L on the accumulation of LC3A/B and mTOR in the presence or absence of Rapa or HCQ. As shown in Figure 4B, the LC3A/B II/I ratio was decreased and the level of P-mTOR was increased in NEDD4L-KD MM cells, suggesting that autophagy was suppressed by low NEDD4L expression. Treatment with Rapa did not further decrease the LC3A/B II/I ratio beyond the effect of NEDD4L inhibition. In contrast, treatment with HCQ, which is used to block LC3A/B-II degradation, resulted in a significant increase in the level of P-mTOR and a decrease in the LC3A/B II/I ratio in NEDD4L-KD cells compared with control cells. Furthermore, overall autophagic fluxes were quantitated in standard assays using NH4Cl to measure the rate of lysosomal digestion of LC3A/B-II as a proxy of autophagic activity, and the LC3A/B II/I ratio was decreased in NEDD4L-KD MM cells (SupFig 2G). These results indicated that NEDD4L may act upstream of mTOR to promote autophagy.

A Cyto-ID Autophagy Detection Kit was then used with flow cytometry to examine the degree of autophagy induced in MM cells after NEDD4L KD. We transfected MM cells with siRNA instead of the lentivirus expressing shRNAs plus GFP to avoid contamination by green fluorescence (data not shown). The results showed a significant shift in the peak fluorescence intensities to lower values in MM cells with NEDD4L inhibition, indicating decreased accumulation of autophagosomes (Figure 4C). Additionally, immunofluorescence analysis revealed a significant decrease in the level of LC3A/B-II in NEDD4L-KD cells treated with HCQ (Figure 4D). Further confirmation of autophagy inhibition was obtained by electron microscopy. NEDD4L KD markedly and significantly (P < 0.05) decreased the number of identified autophagic vacuoles (AVs) per cell following 24 h incubation in HCQ (Figure 4E). All the above data suggested that autophagy was inhibited after NEDD4L KD.

Then, we explored the effect of NEDD4L-regulated autophagy on Bor resistance. Using flow cytometry, we found that rapamycin (Rapa), which is an mTOR inhibitor that activates autophagy, increased the death of MM cells transfected with NEDD4L shRNA in a dose-dependent manner compared to the control group (Figure 4F). Rapa abrogated the protective effect of low NEDD4L against MM cell apoptosis induced by Bor (as seen by comparing the second and fourth columns in Figure 4G). Surprisingly, HCQ, which is an autophagic flux inhibitor, did not affect NEDD4L-mediated differences in MM cell resistance, and no synergistic effect between Bor and HCQ was observed (Figure 4G). The latter result was approximately consistent with that of Jarauta, who also reported that the combination of HCQ with low doses of carfilzomib strongly potentiated apoptosis, but the effect was not observed when HCQ was combined with Bor^21^. The above results suggested that autophagy has the capacity to partially rescue Bor resistance in NEDD4L-KD MM cells.

**NEDD4L limits** **proteasome function and interacts with the proteasome**

Since MM cells are sensitive to imbalances in protein homeostasis, we sought to determine whether NEDD4L impacts the UPS. As chymotrypsin-like (CT-L) enzymatic activity partially represents proteasome activity and is the primary target of Bor, we therefore examined the CT-L peptidase activity of the proteasome in MM cell lines. The expression level of NEDD4L did not correlate with the degradation of CT-L peptides or Bor sensitivity in HMCLs (Figure 5A). However, degradation of CT-L peptides was increased in NEDD4L-KD cells (Figure 5B), indicating that NEDD4L inhibited proteasome activity. Consistent with the increased proteasome activity seen in NEDD4L-KD cells, the level of polyubiquitin conjugates was markedly decreased, and the established UPS target protein phospho-IκBα was significantly increased in these cells (Figure 5C), suggesting that low NEDD4L expression promoted proteasome-dependent protein degradation.

As NEDD4-1 is an E3 ligase, we next identified the substrates related to the proteasome subunits via the UbiBrowser website (SupTable 1) ^22^. We selected five representative substrates and found that 19S proteasome non-ATPase regulatory subunit 2 (PSMD2) was increased in NEDD4L-KD cells in either the presence or absence of Bor. The impact of NEDD4L expression on proteasome-mediated degradation did not appear to be due to quantitative differences in the other four proteasome subunits (Figure 5D). To determine whether NEDD4L interacts with proteasome subunits, we performed a co-IP assay. Figure 5E shows that PSMD2 was present in the NEDD4L complex but not in the IgG control immunoprecipitate, whereas the other four proteasome subunits showed no binding with NEDD4L. Consistently, PSMD2 co-IP indicated NEDD4L presence. Furthermore, we examined more representative 20S and 19S subunits, such as PSMB5, PSMA2, and PSMC3, by immunoblotting. Surprisingly, the protein level of PSMC3, as another 19S regulatory subunit, was increased in NEDD4L-KD cells in the presence of Bor, and NEDD4L interacted with PSMC3. Consistently, PSMC3 co-IP indicated NEDD4L presence (Figure 5F). (**Answers to Reviewer #1 Q5**) However, the protein level of PSMD2 and PSMC3 didn’t change in other NEDD4L-KD turmor cells such as hepatocellular carcinoma (HCC) cells (SupFig 2H). (**Answers to Reviewer #1 Q4**) Taken together, the above data suggested that NEDD4L regulated proteasome activity and proteasome subunit expression and interacted with the 19S proteasome in MM.

**NEDD4L knockdown promotes xenograft tumor growth**

We next evaluated the therapeutic potential of NEDD4L-targeted shRNA using an ARP-1 xenograft NOD/SCID mouse model. Mice were treated with Bor or an equal volume of PBS every three to four days when the tumors reached approximately 100-130 mm^3^ and sacrificed when the tumor size reached approximately 3000 mm^3^. The tumor size was significantly increased in the NEDD4L-KD group compared to the control group on day 28 (Figure 6A and B). No significant change in body weight was observed during the treatment period. Furthermore, the immunohistochemical analysis results shown in Figure 6C indicate that the level of TUNEL was decreased and the level of Ki67 was increased in the NEDD4L-KD group. The above results demonstrated that NEDD4L KD decreased the sensitivity of MM cells to Bor in vivo.

**Discussion**

In this study, we investigated the role of NEDD4L in MM cells and its underlying molecular mechanisms, with a focus on cell death, the cellular process of autophagy and proteasome activity. Our results showed that low NEDD4L expression in MM was associated with poorer response to Bor, advanced disease stage and shorter patient survival times. MM cells with NEDD4L KD exhibited enhanced proliferation and reduced sensitivity to Bor in vitro and in a human MM xenograft SCID mouse model. We further showed that NEDD4L regulated the level of autophagy and proteasome activity in MM cells and that NEDD4L KD in MM cells inhibited autophagy and increased proteasome activity. A working model is schematically represented in Figure 6D.

The expression, functions and substrate specificity of NEDD4L are regulated by deubiquitination, the binding of adaptors and accessory proteins to specific regions of the ligase and intramolecular interactions^23-26^. By western blotting and immunofluorescence assays, NEDD4L was determined to localize mainly in the cytoplasm of MM cells, which is consistent with the results of previous studies^27^. The expression level of NEDD4L decreased as the concentration of Bor increased. We first speculated that the decrease in NEDD4L expression may have been due to changes in protein localization; however, NEDD4L expression decreased in both the cytoplasm and the nucleus of Bor-treated MM cells, which was the same pattern exhibited by NEDD4-1^16^. (**Answers to Reviewer #2 Q3**) Current evidence has revealed that NEDD4-1 can be cleaved during apoptosis and that this cleavage is inhibited by an inhibitor of caspase-3-like proteases^16,19^. Thus, we added three inhibitors of apoptosis to Bor-treated MM cells. Surprisingly, the decrease in NEDD4L expression caused by Bor was abolished by NQDI, as an ASK1 inhibitor, but not by pancaspase inhibitors or Baxi. NQDI mainly regulates the oxidative stress response by inhibiting the expression of ASK1 and its downstream targets, thereby inhibiting cell injury and apoptosis. The above results suggest that the decrease in NEDD4L caused by Bor is not related to caspase- or bax-mediated apoptosis but may be related to increased oxidative stress caused by Bor. The specific causes need further investigation. (**Answers to Reviewer #1 Q2**)

As a ubiquitin ligase, NEDD4L ubiquitinates substrates for recycling, degradation and stabilization via posttranslational modification, and the pathophysiological importance of NEDD4L has gradually become clear from studies on genetically modified mice and single-nucleotide polymorphisms (SNPs)^12^. Previous studies have demonstrated that low NEDD4L expression in non-small cell lung cancer, gastric cancer and gliomas is correlated with poor prognoses, while NEDD4L overexpression promotes melanoma tumor growth^28-32^. Our group first identified NEDD4L as a novel tumor suppressor gene in MM and, by analysis of an Affymetrix HTA 2.0 array, found that NEDD4L is expressed at low levels in Bor-resistant MM cells. In addition, low NEDD4L expression in MM patients correlated with poorer outcomes.

Outcomes of patients with MM are dismal and have not changed for decades. One of the important reasons for MM resistance is mutations in key genes, such as MAF, RAS, and TP53^33^. Our studies found that NEDD4L KD enhanced Bor resistance both in vitro and in vivo. As the UPS and the lysosomal pathway play an important role in both protein degradation and Bor efficacy^34^, we determined the levels of autophagy and proteasome activity in NEDD4L-KD MM cells and compared them with those in control cells. As shown in Figures 4 and 5, autophagy was inhibited while proteasome activity was enhanced by NEDD4L KD. Furthermore, autophagy activators such as Rapa attenuated Bor resistance mediated by low NEDD4L expression. In some cases, cell death can occur due to uncontrolled autophagy in MM^35,36^. Consistent with this observation, Wu *et al.* reported that MIR145-3p exerted a tumor-suppressive function in MM by inducing autophagic cell death and enhancing Bor sensitivity^37^. In addition, 14-3-3 proteins form a complex with NEDD4L^38^ and bind to the proteasome to modulate proteasome activity and assembly^39^. Thus, we further believe that NEDD4L can regulate proteasome activity and thereby affect Bor sensitivity.

Our findings have great clinical value. The proteasomal inhibitor Bor, which inhibits the CTL activity of PSMB5, is now universally used in MM patients. However, resistance and side effects with increased clinical use of Bor have hampered its clinical applications^40^. Therefore, the exploration and identification of markers of Bor sensitivity and methods to increase drug sensitivity are important research topics. We found that the NEDD4L E3 ligase mediated the sensitivity of MM cells to only proteasomal inhibitors (including bortezomib, carfilzomib and ixazomib, SupFig 2C and 2D) and not other drugs, such as lenalidomide, doxorubicin and melphalan (SupFig 2E and 2F), suggesting that NEDD4L expression could be used to identify patients most likely to benefit from PI-based therapy.

In conclusion, our investigation clarified that NEDD4L exerts tumor-suppressive activity in MM, similar to its homolog NEDD4-1, as we reported previously^16^. This E3 ligase functions by enhancing MM cell autophagy and decreasing proteasome activity. Our finding that low expression of NEDD4L attenuated the anti-MM activity of Bor both in vitro and in vivo provides a biological rationale for the use of a NEDD4L activator in combination with Bor as a novel therapeutic strategy for the treatment of multiple myeloma. Additionally, NEDD4L may be a new marker useful for evaluating Bor sensitivity in MM.

**Acknowledgments**

The authors thank Dr. Qing Yi for kindly providing the cells and Ms. Beibei Wang at the Center of Cryo-Electron Microscopy (CCEM) of Zhejiang University for technical assistance with transmission electron microscopy. This work was supported by the National Natural Science Foundation of China (Project No. 81872322, 81901690, and No. 82100213), the National Major Scientific and Technological Special Project for "Significant New Drug Development" 2018ZX09733-003, and the Natural Science Foundation of Zhejiang Province (Project No. YQ20H310012).

**Authorship contributions**

XH, EFZ and WC performed the research; JC and WC analyzed the data; YL, JWQ, YL and XYH contributed to data interpretation; and XH, JSH, HH and ZC wrote the manuscript and designed the experiment.

**Conflict of interest statement**

The authors declare no competing financial interests.

**Data availability**

The data that support the findings of our study are present in the paper. Further details are included in the supplementary methods and reagents or available from the corresponding author upon reasonable request.

**References**

1 Kumar, S. K. *et al.* Multiple myeloma. *Nat Rev Dis Primers* **3**, 17046, doi:10.1038/nrdp.2017.46 (2017).

2 Bazarbachi, A. H., Al Hamed, R., Malard, F., Harousseau, J. L. & Mohty, M. Relapsed refractory multiple myeloma: a comprehensive overview. *Leukemia*, doi:10.1038/s41375-019-0561-2 (2019).

3 Ito, S. Proteasome Inhibitors for the Treatment of Multiple Myeloma. *Cancers (Basel)* **12**, doi:10.3390/cancers12020265 (2020).

4 Lemberg, M. K. & Strisovsky, K. Maintenance of organellar protein homeostasis by ER-associated degradation and related mechanisms. *Mol Cell* **81**, 2507-2519, doi:10.1016/j.molcel.2021.05.004 (2021).

5 Dale, B. *et al.* Advancing targeted protein degradation for cancer therapy. *Nat Rev Cancer*, doi:10.1038/s41568-021-00365-x (2021).

6 Lee, A. H., Iwakoshi, N. N., Anderson, K. C. & Glimcher, L. H. Proteasome inhibitors disrupt the unfolded protein response in myeloma cells. *Proc Natl Acad Sci U S A* **100**, 9946-9951, doi:10.1073/pnas.1334037100 (2003).

7 Jagannathan, S. *et al.* Pharmacologic screens reveal metformin that suppresses GRP78-dependent autophagy to enhance the anti-myeloma effect of bortezomib. *Leukemia* **29**, 2184-2191, doi:10.1038/leu.2015.157 (2015).

8 Bianchi, G. *et al.* The proteasome load versus capacity balance determines apoptotic sensitivity of multiple myeloma cells to proteasome inhibition. *Blood* **113**, 3040-3049, doi:10.1182/blood-2008-08-172734 (2009).

9 Oerlemans, R. *et al.* Molecular basis of bortezomib resistance: proteasome subunit beta5 (PSMB5) gene mutation and overexpression of PSMB5 protein. *Blood* **112**, 2489-2499, doi:10.1182/blood-2007-08-104950 (2008).

10 Barrio, S. *et al.* Spectrum and functional validation of PSMB5 mutations in multiple myeloma. *Leukemia* **33**, 447-456, doi:10.1038/s41375-018-0216-8 (2019).

11 Vogl, D. T. *et al.* Combined autophagy and proteasome inhibition: a phase 1 trial of hydroxychloroquine and bortezomib in patients with relapsed/refractory myeloma. *Autophagy* **10**, 1380-1390, doi:10.4161/auto.29264 (2014).

12 Manning, J. A. & Kumar, S. Physiological Functions of Nedd4-2: Lessons from Knockout Mouse Models. *Trends Biochem Sci* **43**, 635-647, doi:10.1016/j.tibs.2018.06.004 (2018).

13 Wang, H. *et al.* Endoplasmic reticulum stress up-regulates Nedd4-2 to induce autophagy. *FASEB J* **30**, 2549-2556, doi:10.1096/fj.201500119 (2016).

14 Nazio, F. & Cecconi, F. Autophagy up and down by outsmarting the incredible ULK. *Autophagy* **13**, 967-968, doi:10.1080/15548627.2017.1285473 (2017).

15 Huang, X. *et al.* The many substrates and functions of NEDD4-1. *Cell Death Dis* **10**, 904, doi:10.1038/s41419-019-2142-8 (2019).

16 Huang, X. *et al.* The NEDD4-1 E3 ubiquitin ligase: A potential molecular target for bortezomib sensitivity in multiple myeloma. *Int J Cancer*, doi:10.1002/ijc.32615 (2019).

17 Yang, L. *et al.* Pirh2 mediates the sensitivity of myeloma cells to bortezomib via canonical NF-kappaB signaling pathway. *Protein Cell* **9**, 770-784, doi:10.1007/s13238-017-0500-9 (2018).

18 Weissman, A. M., Shabek, N. & Ciechanover, A. The predator becomes the prey: regulating the ubiquitin system by ubiquitylation and degradation. *Nat Rev Mol Cell Biol* **12**, 605-620, doi:10.1038/nrm3173 (2011).

19 Harvey, K. F. *et al.* Caspase-mediated cleavage of the ubiquitin-protein ligase Nedd4 during apoptosis. *J Biol Chem* **273**, 13524-13530, doi:10.1074/jbc.273.22.13524 (1998).

20 Qian, W. *et al.* Wogonin Attenuates Isoprenaline-Induced Myocardial Hypertrophy in Mice by Suppressing the PI3K/Akt Pathway. *Front Pharmacol* **9**, 896, doi:10.3389/fphar.2018.00896 (2018).

21 Jarauta, V. *et al.* Inhibition of autophagy with chloroquine potentiates carfilzomib-induced apoptosis in myeloma cells in vitro and in vivo. *Cancer Lett* **382**, 1-10, doi:10.1016/j.canlet.2016.08.019 (2016).

22 Li, Y. *et al.* An integrated bioinformatics platform for investigating the human E3 ubiquitin ligase-substrate interaction network. *Nat Commun* **8**, 347, doi:10.1038/s41467-017-00299-9 (2017).

23 Oberfeld, B. *et al.* Ubiquitin-specific protease 2-45 (Usp2-45) binds to epithelial Na+ channel (ENaC)-ubiquitylating enzyme Nedd4-2. *Am J Physiol Renal Physiol* **301**, F189-196, doi:10.1152/ajprenal.00487.2010 (2011).

24 Konstas, A. A. *et al.* Regulation of the epithelial sodium channel by N4WBP5A, a novel Nedd4/Nedd4-2-interacting protein. *J Biol Chem* **277**, 29406-29416, doi:10.1074/jbc.M203018200 (2002).

25 Yip, K. H. *et al.* The Nedd4-2/Ndfip1 axis is a negative regulator of IgE-mediated mast cell activation. *Nat Commun* **7**, 13198, doi:10.1038/ncomms13198 (2016).

26 Bruce, M. C. *et al.* Regulation of Nedd4-2 self-ubiquitination and stability by a PY motif located within its HECT-domain. *Biochem J* **415**, 155-163, doi:10.1042/BJ20071708 (2008).

27 Garrone, N. F., Blazer-Yost, B. L., Weiss, R. B., Lalouel, J. M. & Rohrwasser, A. A human polymorphism affects NEDD4L subcellular targeting by leading to two isoforms that contain or lack a C2 domain. *BMC Cell Biol* **10**, 26, doi:10.1186/1471-2121-10-26 (2009).

28 Sakashita, H. *et al.* Identification of the NEDD4L gene as a prognostic marker by integrated microarray analysis of copy number and gene expression profiling in non-small cell lung cancer. *Ann Surg Oncol* **20 Suppl 3**, S590-598, doi:10.1245/s10434-013-3059-6 (2013).

29 Jiang, X. *et al.* The correlation between NEDD4L and HIF-1alpha levels as a gastric cancer prognostic marker. *Int J Med Sci* **16**, 1517-1524, doi:10.7150/ijms.34646 (2019).

30 Gao, C., Pang, L., Ren, C. & Ma, T. Decreased expression of Nedd4L correlates with poor prognosis in gastric cancer patient. *Med Oncol* **29**, 1733-1738, doi:10.1007/s12032-011-0061-3 (2012).

31 He, S. *et al.* Down-regulation of Nedd4L is associated with the aggressive progression and worse prognosis of malignant glioma. *Jpn J Clin Oncol* **42**, 196-201, doi:10.1093/jjco/hyr195 (2012).

32 Kito, Y. *et al.* Pathobiological properties of the ubiquitin ligase Nedd4L in melanoma. *Int J Exp Pathol* **95**, 24-28, doi:10.1111/iep.12051 (2014).

33 Papadas, A. & Asimakopoulos, F. Mechanisms of Resistance in Multiple Myeloma. *Handb Exp Pharmacol* **249**, 251-288, doi:10.1007/164_2017_10 (2018).

34 Cao, B. & Mao, X. The ubiquitin-proteasomal system is critical for multiple myeloma: implications in drug discovery. *Am J Blood Res* **1**, 46-56 (2011).

35 Cao, B. *et al.* Clioquinol induces pro-death autophagy in leukemia and myeloma cells by disrupting the mTOR signaling pathway. *Sci Rep* **4**, 5749, doi:10.1038/srep05749 (2014).

36 Lamy, L. *et al.* Control of autophagic cell death by caspase-10 in multiple myeloma. *Cancer Cell* **23**, 435-449, doi:10.1016/j.ccr.2013.02.017 (2013).

37 Wu, H. *et al.* MIR145-3p promotes autophagy and enhances bortezomib sensitivity in multiple myeloma by targeting HDAC4. *Autophagy*, 1-15, doi:10.1080/15548627.2019.1635380 (2019).

38 Ho, P. Y. *et al.* beta1Pix exchange factor stabilizes the ubiquitin ligase Nedd4-2 and plays a critical role in ENaC regulation by AMPK in kidney epithelial cells. *J Biol Chem* **293**, 11612-11624, doi:10.1074/jbc.RA118.003082 (2018).

39 Gu, Y. *et al.* 14-3-3zeta binds the proteasome, limits proteolytic function and enhances sensitivity to proteasome inhibitors. *Leukemia* **32**, 744-751, doi:10.1038/leu.2017.288 (2018).

40 Wallington-Beddoe, C. T., Sobieraj-Teague, M., Kuss, B. J. & Pitson, S. M. Resistance to proteasome inhibitors and other targeted therapies in myeloma. *Br J Haematol* **182**, 11-28, doi:10.1111/bjh.15210 (2018).

**Figure legends**

**Figure 1. Clinical significance of NEDD4L in MM. (**A) Heatmap of the Affymetrix HTA 2.0 array in RPMI8226 and RPMI8226.BR cells showing the expression of 14 E3s. (B-F) Boxplots of log2 NEDD4L gene expression based on Oncomine data. NEDD4L expression in patients with different Bor responses (NR, n=84; RES, n=85) (B) and progression (NO, n=53; YES, n=189) (C). NEDD4L expression in plasma cells purified from tissues at different stages of MM and normal tissues (NPCs, n=5; MGUS, n=11; MM, n=133; PCL, n=9) (D). Overall survival status according to NEDD4L expression (Alive, n=48, Dead, n=16) (E). NEDD4L expression in patients without recurrence (No-R, n=38) and with recurrence (REC, n=26) (F). No value indicates patients without collected status results. (G) Immunohistochemical staining of NEDD4L in BM biopsies from healthy donors (HD) and relapsed/refractory MM patients (RRMM). Three representative samples are shown. Scale bars, 50 μm. (H) qPCR assay of NEDD4L expression in NDMM and RRMM. (**Answers to Reviewer #2 Q2**)

**Figure 2. NEDD4L expression in MM.** (A) Western blotting and qPCR assays of NEDD4L expression in human MM cell lines and PBMCs. (B) Immunofluorescence staining analysis of the subcellular localization of NEDD4L in CD138^+^ cells from BM biopsy tissues of MM patients. Nuclei were stained with DAPI. Scale bars, 50 μm. (C) Western blotting and qPCR analyses of NEDD4L expression in human MM cell lines treated with different concentrations of Bor (0, 5, 10, and 20 nM) for 24 h. (D) Western blotting analysis of the subcellular localization of NEDD4L in HMCLs treated with or without Bor (10 nM). Cyto refers to the cytoplasm, and Nuc refers to the nucleus. (E) ARP-1 and RPMI8226 cells were treated with or without Bor (10 nM), NQDI-1 (15 μM), Baxi (15 μM), or Z-VAD-FMK (40 μM). Whole-cell extracts were analyzed by western blotting with anti-NEDD4L and anti-GAPDH antibodies. NQDI-1: an ASK1 inhibitor, Baxi: a Bax-mediated apoptosis inhibitor.

**Figure 3. NEDD4L regulates the Bor sensitivity in MM cells.** (A) Western blotting and qPCR analyses showing the expression of NEDD4L in NEDD4L-KD and control cells. “ns” means “nonsignificant”. (B) CCK-8 cell proliferation assay in cell lines with stable NEDD4L KD with or without exposure to 3 nM Bor or 1% FBS for 5 days. (C, D) Cell apoptosis was detected by CCK-8 and flow cytometry in cell lines with stable NEDD4L KD treated with the indicated concentration of Bor for 24 h. (E) Western blotTING analysis showing the expression of NEDD4L in NEDD4L-overexpressing and control cells. Cell apoptosis was detected by CCK-8 and flow cytometry in cell lines with stable NEDD4L overexpression treated with the indicated concentration of Bor for 24 h. (F) Cell cycle assay performed by flow cytometry showing the percentages of cells in G1, S, and G2 phase in NEDD4L-KD cells treated with or without Bor for 24 h. The histograms show the percentages of HMCLs in G_1_, S, and G_2_ phase in three independent experiments. (G) Western blot assay of NEDD4L and P21 in cells with stable NEDD4L KD after treatment with 10 nM Bor for 24 h.

**Figure 4. An autophagy activator attenuates Bor resistance elicited by low NEDD4L expression in vitro.** (A) Western blot assay of PI3K/AKT/mTOR expression levels in cell lines with stable NEDD4L KD. GAPDH was used as the loading control. (B) Western blot assay of NEDD4L, LC3A/B, mTOR, and P-mTOR in cells with stable NEDD4L KD after treatment with Rapa or HCQ for 24 h. (C) Autophagosome formation upon treatment of ARP-1 cells with or without HCQ. MM cells (3 × 10^5^ cells/ml) were incubated with HCQ (10 μM) for 24 h, labeled with the Cyto-ID probe and analyzed by flow cytometry. (D) Immunofluorescence staining analysis of LC3A/B-II expression in the presence of HCQ (10 μM) in ARP-1 cells. Nuclei were stained with DAPI. Magnification: 200×. (E) Representative electron micrographs of ARP-1 MM cells treated with HCQ (10 μM) for 24 h. Red arrows show areas of AVs. Magnification: 2 μm. The right histogram shows the quantification of AVs. (F) Flow cytometric apoptosis assay in cells with stable NEDD4L KD treated with Bor and increasing doses of Rapa for 24 h. The histograms show the percentages of cells undergoing apoptosis. “ns” means “nonsignificant”. (G) Cell apoptosis detected by flow cytometry in cell lines with stable NEDD4L KD treated with Bor, Rapa or HCQ for 24 h. The histograms show the percentages of cells undergoing apoptosis. “ns” means “nonsignificant”.

**Figure 5. NEDD4L regulates proteasome activity and interacts with the 19S proteasome.** (A) NEDD4L expression, proteasome enzymatic activity and Bor sensitivity in 5 HMCLs. A proteasome CTL activity assay was conducted using Suc-LLVY-AMC as a substrate in extracts from MM cell lines. MG132 (1 μM) was used as the positive control. Data were collected at 37°C every 30 minutes. Cell viability was detected by a CCK-8 assay in HMCLs treated with the indicated concentration of Bor for 24 h. (B) Effect of NEDD4L on proteasome enzymatic activity in MM cell lines with stable NEDD4L KD. (C) Western blot assay of polyubiquitin conjugates and IkBα and phospho-IkBα levels in cell lines with stable NEDD4L KD in the presence or absence of Bor. GAPDH was used as the loading control. (D) Western blot assay of proteasome subunit expression levels in cell lines with stable NEDD4L KD. GAPDH was used as the loading control. (E) NEDD4L immunoprecipitated with endogenous PSMD2 and PSMD2 reversely co-IP with NEDD4L. HMCL extracts were subjected to immunoprecipitation with antibodies against NEDD4L or PSMD2 along with the IgG control antibody and then to western blotting with specific antibodies for proteasome subunit proteins. (F) NEDD4L immunoprecipitated with representative 20S and 19S subunits, and PSMC3 reversely co-IP with NEDD4L. (**Answers to Reviewer #1 Q5**) Western blot assay of PSMC3 in cell lines with stable NEDD4L KD. GAPDH was used as the loading control.

**Figure 6. NEDD4L KD decreased the sensitivity of MM cells to Bor in vivo.** (A, B) Tumor volumes measured by Vernier calipers in the 6 different groups (3 mice per group). (C) Immunohistochemical analysis was performed to detect NEDD4L, HE, Ki67, and TUNEL (magnification: 400×). (D) Schematic model of NEDD4L in MM cells.
