# Supplementary material for: NEDD4L binds the proteasome and promotes autophagy and bortezomib sensitivity in multiple myeloma
Source: Cell Death Dis. 2022 Mar 2;13(3):197. doi: 10.1038/s41419-022-04629-8 (PMC8891287; doi:10.1038/s41419-022-04629-8)

**Supplementary Information**

**Methods and reagents**

**Quantitative RT-PCR assay**

The quantitative RT-PCR assay was performed as previously described. The following primers were used for qRT-PCR: RNF168 (forward, 5’- TCAACGTGGAACTGTGGACG-3’; reverse, 5’-CAGGTTTACTGAGCAGACGAAC-3’), HERC2 (forward, 5’-GCCAAGTCAATTCTGGACAGC-3’; reverse, 5’-CCTCTGCTTGAGTCGCAGG-3’), and

3-Mar (forward, 5’-AGAGCCCCTTCAATGACCG-3’; reverse, 5’- GCAGCTCCGATGAATTGTCC-3’).

**Supplementary Figures 1–5 and legends:**

**SFigure 1. NEDD4L expression varied in MM cells.** (A) q-PCR assays of RNF168, HERC2, NEDD4L, and 3-Mar expression in human MM cell lines. (B) Boxplots of log2 NEDD4L gene expression based on Oncomine data. NEDD4L expression in patients presenting with different bone lesions (Negative, n=55; Positive, n=65). (C) Immunofluorescence analysis of the negative control staining for NEDD4L and CD138 in MM patients. Nuclei were stained with DAPI. Scale bars, 50 μm. (D) ARP-1 cells were treated with Bor (10 nM) or Z-VAD-FMK (40 mM). Whole-cell extracts were analyzed by Western blotting with antibodies against NEDD4L, caspase-3, and GAPDH.

**SFigure 2. NEDD4L knockdown mediated PIs resistance and autophagy in MM cells.** (A) HMCLs with NEDD4L knockdown were treated with the indicated concentration of Bor. After 24 h of incubation at 37°C, apoptosis was detected by flow cytometry. Annexin V^+^ cells were considered apoptotic cells. The histograms show the percentage of cells undergoing apoptosis. “ns” means “nonsignificant”. (B) Cell apoptosis was detected by flow cytometry in cell lines with stable NEDD4L overexpression treated with the indicated concentration of Bor for 24 h. The histograms show the percentage of cells undergoing apoptosis. “ns” means “nonsignificant”. (C-F) HMCLs with NEDD4L knockdown were treated with the indicated concentration of CFZ, IXA, LEN, MEL or ADM. After 24 h of incubation at 37°C, apoptosis was detected by flow cytometry. Annexin V^+^ cells were considered apoptotic cells. The histograms show the percentage of cells undergoing apoptosis. “ns” means “nonsignificant”. (G) Western blot assay of LC3A/B in cells with stable NEDD4L knockdown after treatment with the indicated concentration of NH4CL for 24 h. (H) Western blot assay of PSMD2 and PSMC3 in HCC cells with stable NEDD4L knockdown.

**SFigure 3-5.** Protein bands throughout the manuscript were analyzed with Image Lab software. *P < 0.05, **P < 0.01, ***P < 0.001.


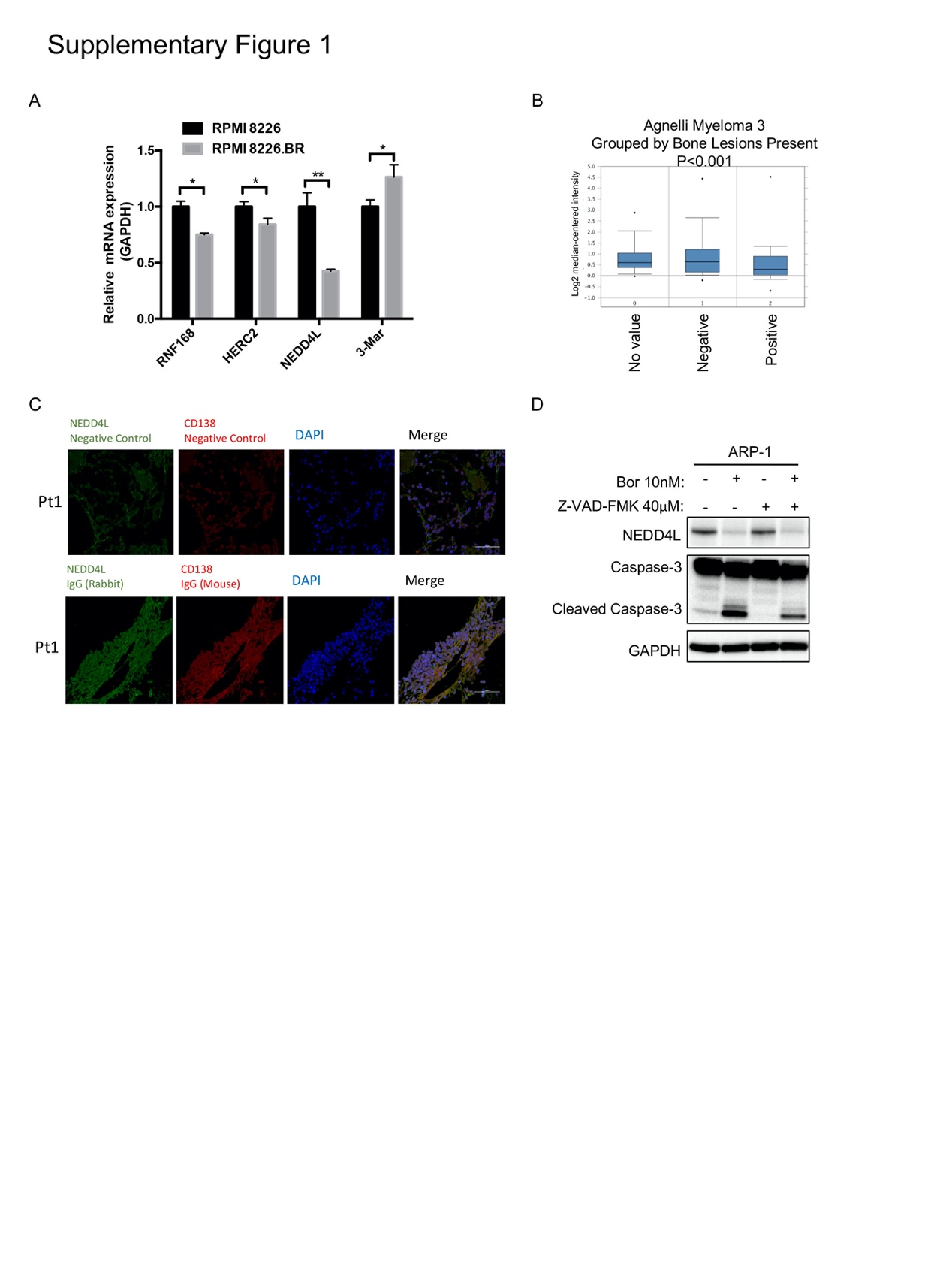

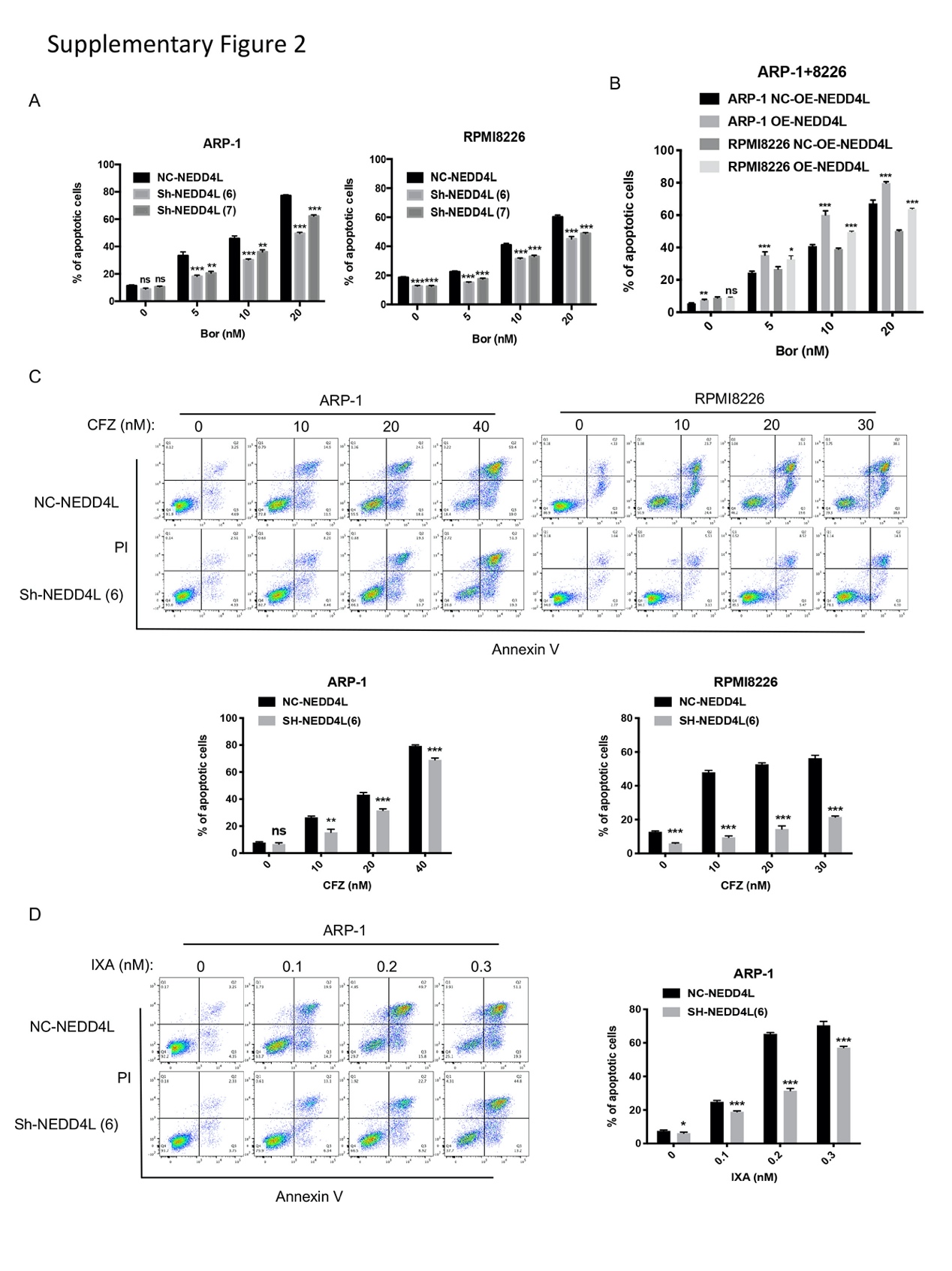


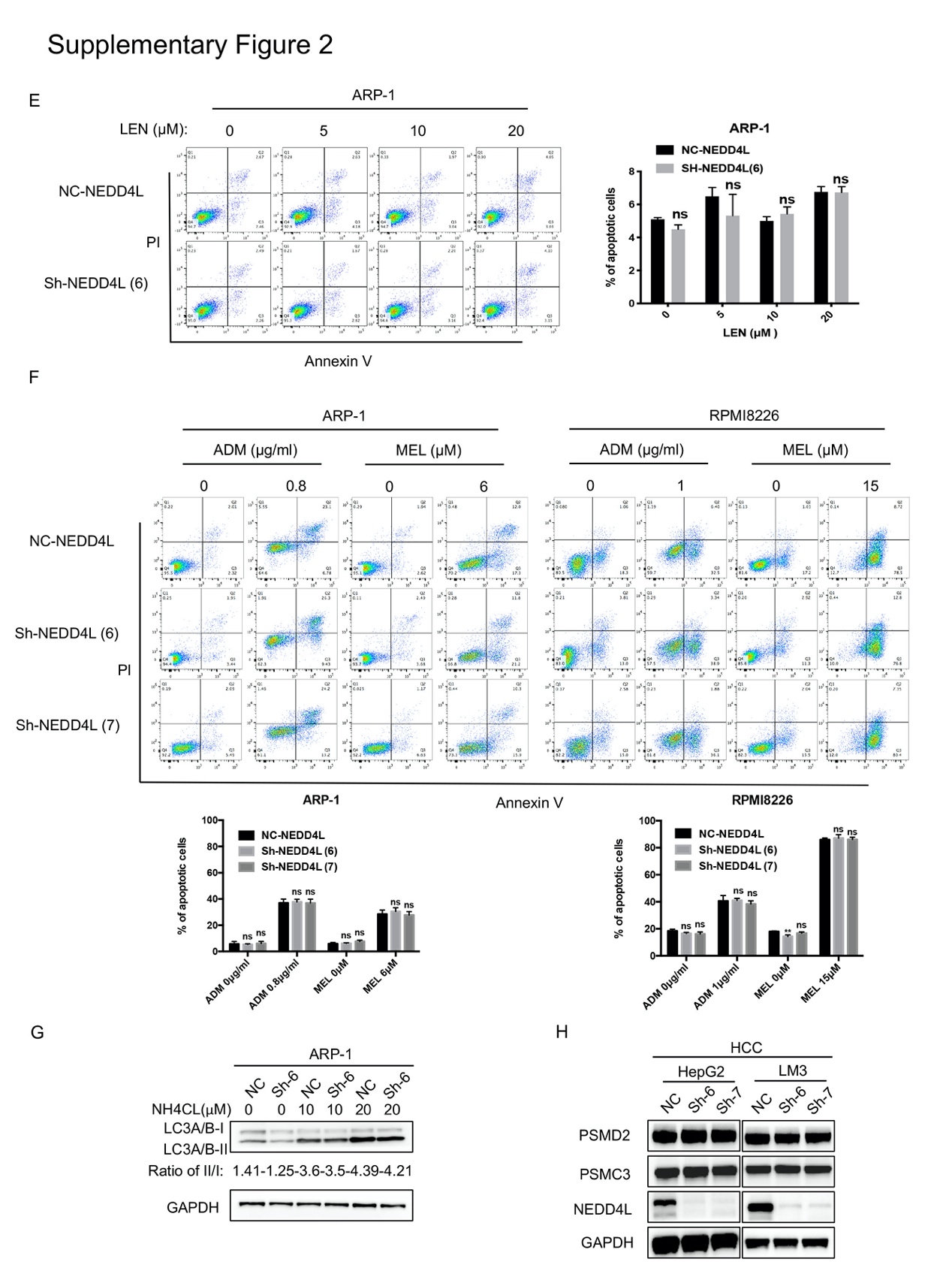


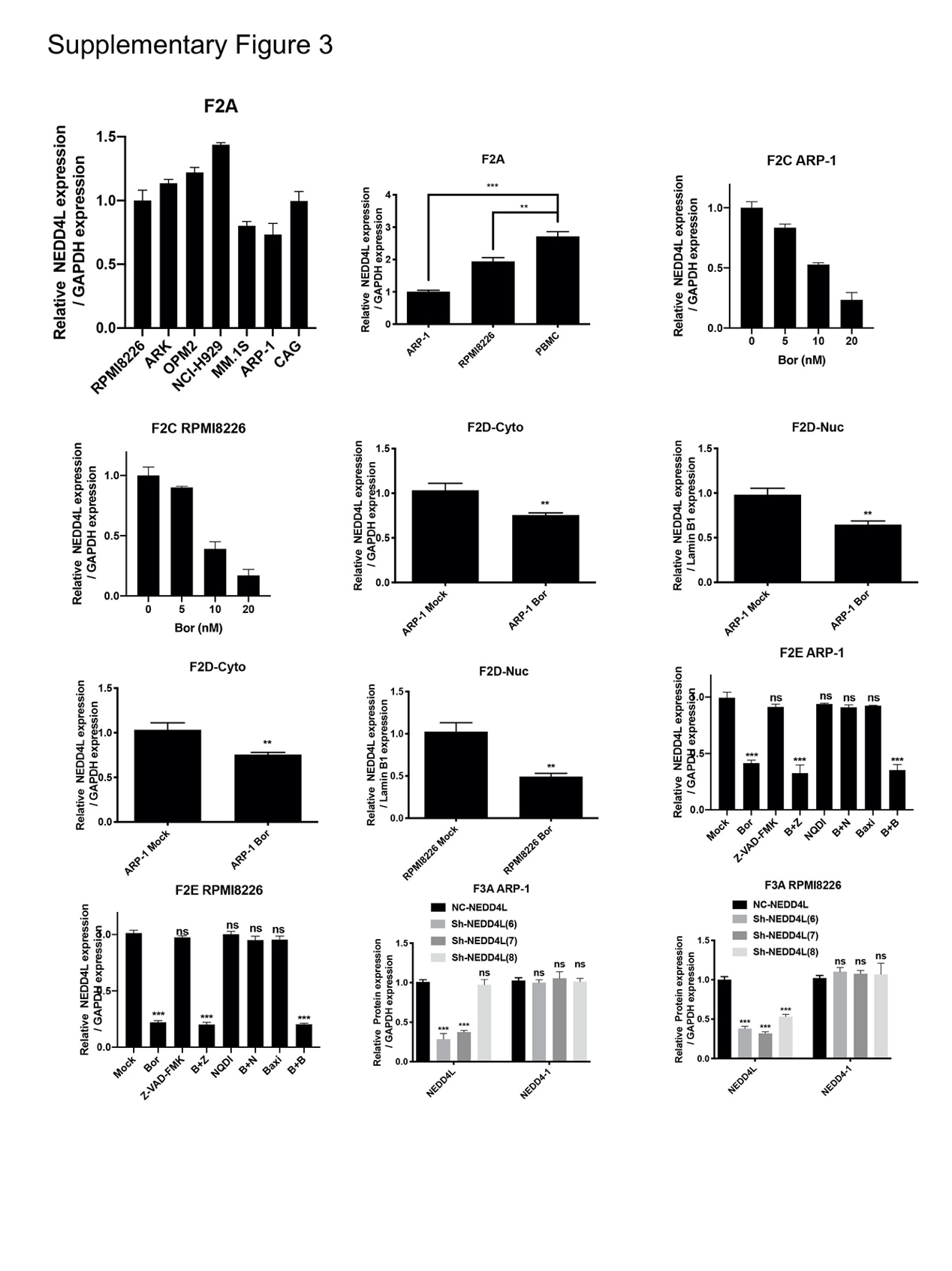


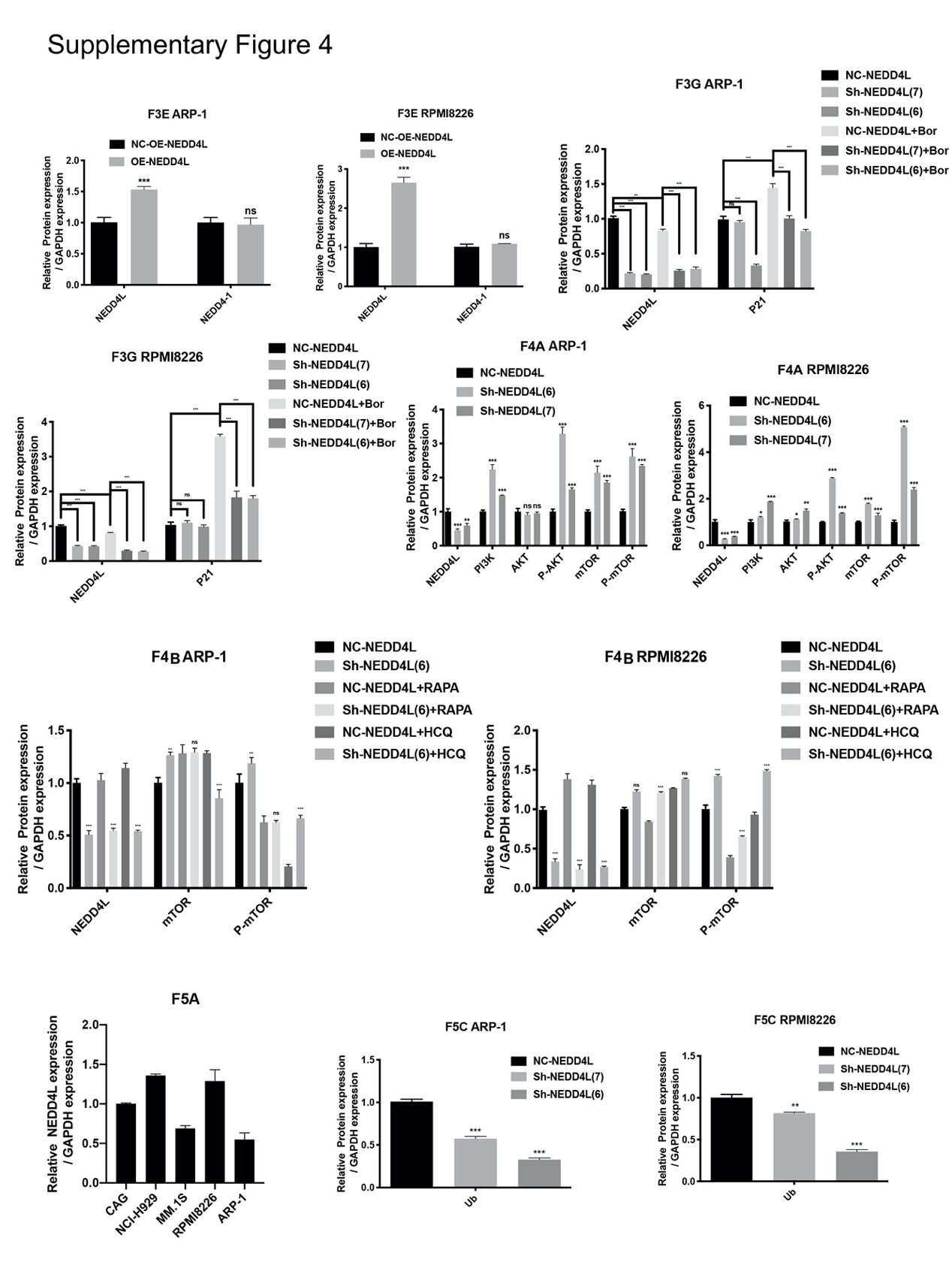


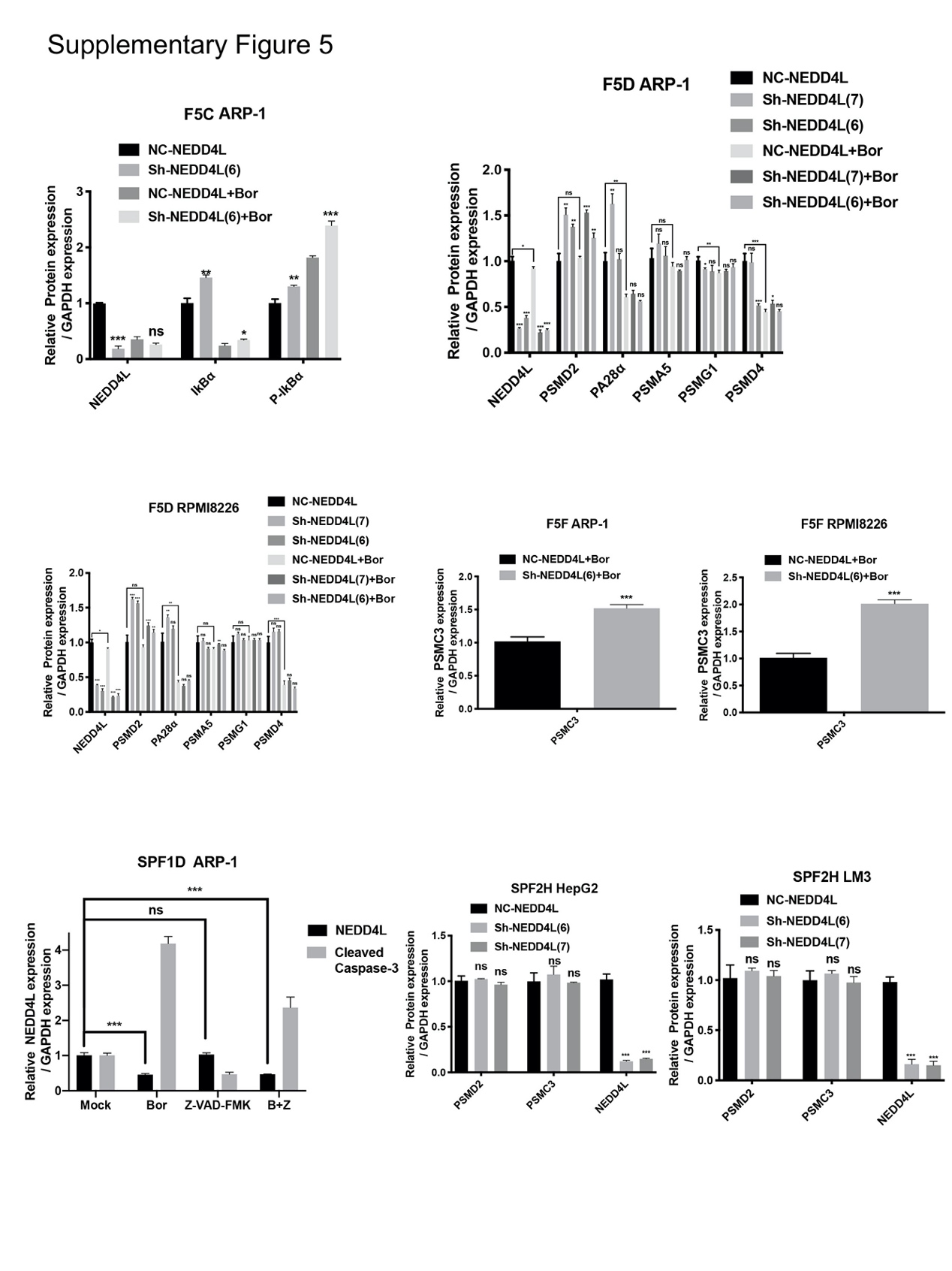

Supplement: Supplementary file 2 — supplementary information [file 41419_2022_4629_MOESM2_ESM.docx]
